# Identification and comprehensive analysis of circRNA–miRNA–mRNA regulatory networks in osteoarthritis

**DOI:** 10.3389/fimmu.2022.1050743

**Published:** 2023-01-09

**Authors:** Xuanzhe Liu, Huimin Xiao, Xiaotong Peng, Yimin Chai, Shuo Wang, Gen Wen

**Affiliations:** ^1^ Department of Orthopedic Surgery, Shanghai Sixth People’s Hospital Affiliated to Shanghai Jiao Tong University School of Medicine, Shanghai, China; ^2^ College of Fisheries and Life Science, Shanghai Ocean University, Shanghai, China; ^3^ Department of Gynecology, Shanghai First Maternity and Infant Hospital, School of Medicine, Tongji University, Shanghai, China

**Keywords:** circular RNA, microRNA, osteoarthritis, chondrocytes, regulatory network

## Abstract

Osteoarthritis (OA) is a common orthopedic degenerative disease, leading to high disability in activities of daily living. There remains an urgent need to identify the underlying mechanisms and identify new therapeutic targets in OA diagnosis and treatment. Circular RNAs (circRNAs) play a role in the development of multiple diseases. Many studies have reported that circRNAs regulate microRNAs (miRNAs) through an endogenous competitive mechanism. However, it remains unclear if an interplay between circRNAs, miRNAs, and target genes plays a deeper regulatory role in OA. Four datasets were downloaded from the GEO database, and differentially expressed circRNAs (DECs), differentially expressed miRNAs (DEMs), and differentially expressed genes (DEGs) were identified. Functional annotation and pathway enrichment analysis of DEGs and DECs were carried out to determine the main associated mechanism in OA. A protein–protein network (PPI) was constructed to analyze the function of, and to screen out, hub DEGs in OA. Based on the artificial intelligence prediction of protein crystal structures of two hub DEGs, TOP2A and PLK1, digitoxin and oxytetracycline were found to have the strongest affinity, respectively, with molecular docking. Subsequently, overlapping DEMs and miRNAs targeted by DECs obtained target DEMs (DETMs). Intersection of DEGs and genes targeted by DEMs obtained target DEGs (DETGs). Thus, a circRNA–miRNA–mRNA regulatory network was constructed from 16 circRNAs, 32 miRNAs, and 97 mRNAs. Three hub DECs have the largest number of regulated miRNAs and were verified through *in vitro* experiments. In addition, the expression level of 16 DECs was validated by RT-PCR. In conclusion, we constructed a circRNA–miRNA–mRNA regulatory network in OA and three new hub DECs, hsa_circ_0027914, hsa_circ_0101125, and hsa_circ_0102564, were identified as novel biomarkers for OA.

## Highlights

1. For the first time, a circRNA–miRNA–mRNA regulatory network, constructed from 16 DECs, 32DETMs, and 97 DETGs, was identified in OA chondrocytes.
2. Three hub DECs, hsa_circ_0027914, hsa_circ_0101125, and hsa_circ_0102564, were identified as the hub DECs with the most regulated miRNAs in OA. *In vitro* validation experiments suggested the pro-apoptosis function of these three hub DECs and their potential to be novel biomarkers in OA.
3. Digitoxin and oxytetracycline have the highest affinity for the target genes, *TOP2A* and *PLK1*, and thus were identified as potential drugs for OA therapy.

## Introduction

Osteoarthritis (OA) is one of the most common long-term degenerative joint diseases, and the leading cause of disability in elderly people (1). Recent epidemiology studies have reported that the incidence rate of knee OA peaks around the age of 75 years, at 16% (2). The main pathophysiological changes of OA include articular cartilage wear and tear, subchondral bone destruction, and synovium inflammation (1). The pathogenesis of OA is multifactorial, and the molecular biological mechanism remains unclear. To date, few biomarkers of OA have been utilized to develop early diagnosis and treatment.

With deeper understanding of the cellular and molecular biology, the role of post-transcriptional modifications of non-coding RNAs (ncRNAs) in the regulation of messenger RNAs (mRNAs) has received increasing attention (3). Non-coding RNAs (ncRNAs) include microRNAs(miRNAs), circular RNAs (circRNAs), long-non-coding RNAs (lncRNAs), etc. Among these, the functions of miRNAs and circRNAs are the most widely studied owing to their paradoxical effect on mRNA regulation (4). miRNAs are single-stranded RNA molecules (about 18–25 nucleotides in length). At the post-transcriptional level, miRNAs can bind to complementary sites on the 3′-untranslated region (3′-UTR) of target genes to negatively regulate mRNA expression (5). circRNAs, in contrast, are competing endogenous RNAs (ceRNAs), characterized by a closed continuous loop structure lacking a 5′ cap and a poly(A) tail at the 3′ ends (4). The main function of circRNAs is to act as a molecular sponge. CircRNAs can target miRNA and inhibit miRNA transcription, thus enhancing expression of the target gene (6).

Recently, increasing evidence has shown that the interaction between miRNAs and circRNAs plays a vital role in the pathogenesis of many human diseases (7), especially osteoarthritis (OA) (8, 9). CircRNA and miRNAs are strongly related to extracellular matrix (ECM) metabolism and inflammation in OA. For instance, circCDK14 (hsa_circ_0001722) has a protective effect on the ECM and can sponge miR-125a-5p by restoring TGF-β/Smad2 pathways in OA (10). In addition, another circPDE4D protects against OA by binding to miR-103a-3p and targeting the fibroblast growth factor 18 (FGF18) gene (11). Both of these studies found that circRNA competes with miRNA. However, the particular miRNA and circRNA involved in in OA and their potential regulatory interplay remained unidentified.

**Scheme 1:**
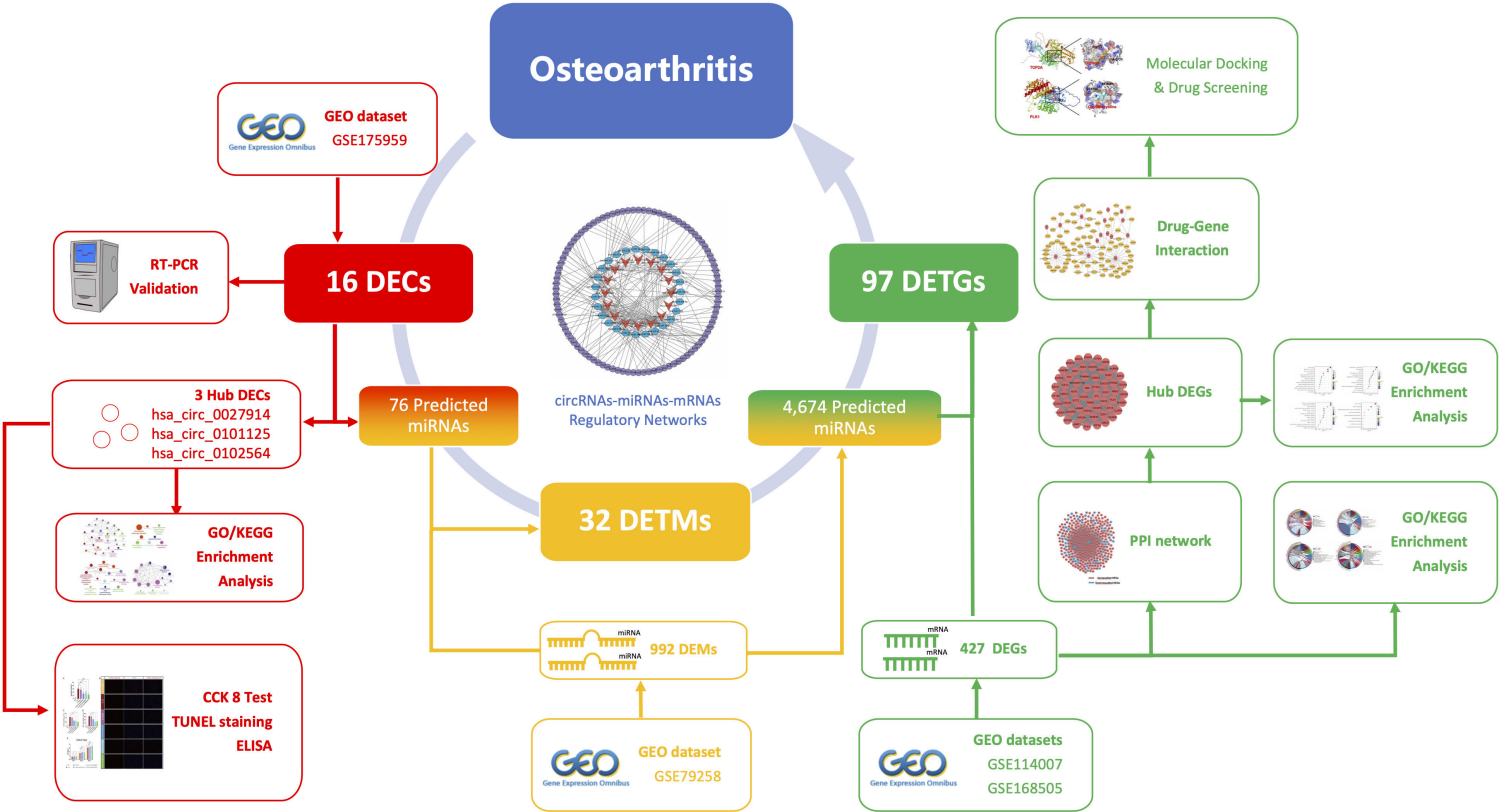
The schematic workflow of this study.

As shown in **Scheme 1**, our study aimed to reveal the novel pathogenic mechanism of circRNAs in OA through bioinformatic analysis, and to identify new potential drug targets. Furthermore, the circRNA–miRNA–mRNA network and target gene–protein interaction network are shown, to illustrate the interplay of downstream mRNAs. We also aimed to validate the function of these pathogenic circRNAs through *in vitro* experiments.

## Materials and methods

### Data derivation

**Figure 1:**
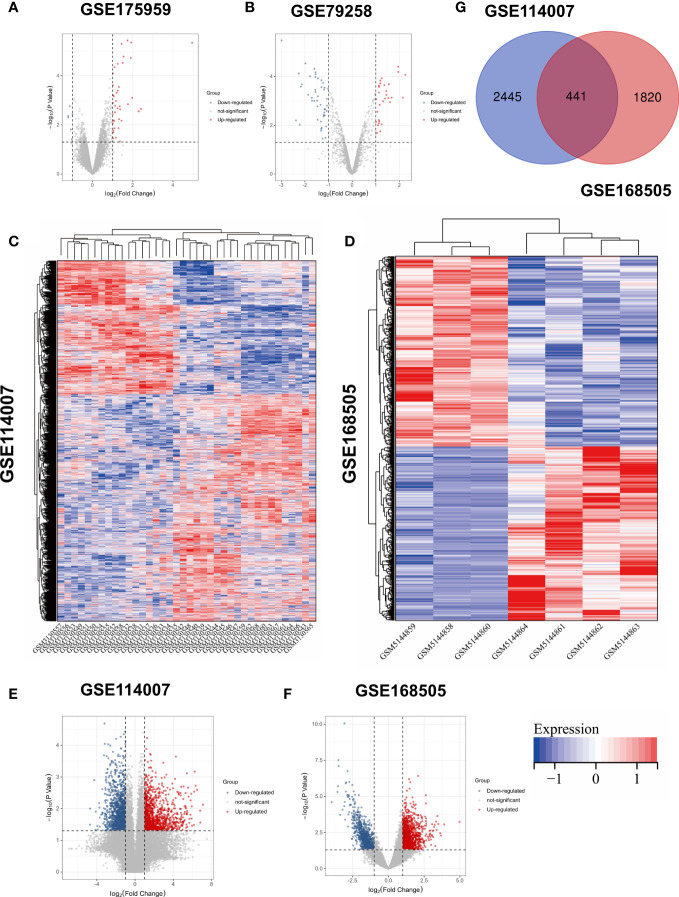
Volcano plots of **(A)** GSE175959; **(B)** GSE 79258; Heatmaps of **(C)** GSE114007; **(D)** GSE168505; Volcano plots of **(E)** GSE114007; **(F)** GSE168505. **(G)** Venn diagram of screening DEGs form mRNA expression profile.

OA-related expression datasets for circRNA (GSE175959), miRNA (GSE79258), and mRNA (GSE114007 and GSE168505) were extracted from the Gene Expression Omnibus (GEO) database (https://www.ncbi.nlm.nih.gov/geo/). The characterization of these datasets is shown in **Figure 1A**. Owing to the batch nature of the GSE data, we integrated and normalized these data using R’s limma package.

### Identification of DECs, DEMs, and DEGs

The pretreated datasets were further analyzed using the GEO2R (http://www.ncbi.nlm.nih.gov/geo/geo2r/) for comparison between OA tissue and matched non-arthritic cartilage tissue. The screening thresholds of differentially expressed genes (DEGs), differentially expressed microRNAs (DEMs), and differentially expressed circular RNAs (DECs) were set as *p*-value < 0.05 and |log_2_-fold change (FC)| > 1. DEGs were divided into two subgroups: upregulated DEGs and downregulated DEGs. Volcano plots were used to visualize DEGs, DEMs, and DECs. Heatmaps were used to better demonstrate the expression profiles of DEGs, and they were created by TBtools software.

### Construction of protein–protein interaction (PPI) network and identification of hub genes

A PPI network was constructed using the online STRING database (http://www.string-db.org/) and Cytoscape, as described previously (12). In addition, hub genes were obtained using molecular complex detection (MCODE) v1.5.1.16 (13) (degree of cut-off = 2, max. depth = 100, node score cut-off = 0.2, and k-core = 2).

### Functional enrichment analysis of DEGs and DECs

Gene Ontology (GO) and Kyoto Encyclopedia of Genes and Genomes (KEGG) pathway functional enrichment analysis were performed in the Sangerbox database (http://sangerbox.com/Tool) to annotate DEGs and hub DEGs. In addition, functional annotation of hub DECs was carried out based on the analysis of target genes regulated by hub DECs. The enrichment results were visualized by ClueGO V2.5.1 (with a *p*-value < 0.05 and a gene count > 3).

### Drug–gene network analysis

Target drugs of hub genes were further investigated using an online drug–gene interaction database (DGIdb, https://dgidb.genome.wustl.edu/). The drug–gene network was then constructed using Cytoscape.

### Homology modeling and molecular docking

With the help of artificial intelligence (AI), protein structural biology has advanced significantly in recent years. AlphaFold v2.0 (https://alphafold.ebi.ac.uk/) was adopted to build the structure of TOP2A and PLK1 using *in silico* modeling as an AI prediction tool. The amino acid sequences of human TOP2A and PLK1 (accession number O15392 and P04818, respectively) were obtained from the UniProt-KB database (http://www.uniprot.org/). The local distance difference test (LDDT) score in the AlphaFold database was used for the evaluation of the stereochemical quality of these predictive models.

After analysis of protein structure, molecular docking helps to mimic the first rank orientation of small-molecule drugs to macromolecules to better understand drug–gene interaction by computational simulation. Related small molecules were obtained from the PubChem database (https://pubchem.ncbi.nlm.nih.gov). AutoDockTools and PyMol were applied to hydrogenate or delete the crystallographic water and ligands of molecular structure. Molecular docking was performed by AutoDock Vina with default parameters. The geometric structures of selected drugs and candidate genes were then visualized. Docking affinity was also scored. PyMol was applied to analyze the optimal docking conformation.

### Prediction of circRNA–miRNA and miRNA–mRNA interactions

CircRNA sponging target miRNAs was predicted by CircBank (http://www.circbank.cn/index.html) (14). The intersection of predicted target miRNAs and DEMs was then considered as differentially expressed target miRNAs (DETMs) for further miRNA–mRNA network construction. Subsequently, miRWalk3.0 (http://zmf.umm.uni-heidelberg.de/apps/zmf/mirwalk3/) was applied to predict target genes of DETMs. Then, differentially expressed target genes (DETGs) were obtained by taking the intersection of the predicted genes and DEGs. Eventually, circRNA–miRNA–target gene regulatory networks were visualized by Cytoscape. Among the DECs in the ceRNA network, we adopted the DECs with the largest number of regulated miRNAs as the top three ranking hub DECs.

### Functional enrichment analysis of hub DECs

To further reveal the function of hub DECs, GO and KEGG pathways analysis of DETGs regulated by hub DECs was performed using ClueGO (https://apps.cytoscape.org/apps/cluego). An adjusted *p*-value of < 0.05 was considered to indicate significant enrichment.

### Tissue samples collection

Five samples of human arthritic cartilage and five samples of human non-arthritic cartilage were collected from patients who had undergone knee arthroplasty surgery in Shanghai Jiao Tong University Affiliated Sixth People’s Hospital. All procedures were approved by the Ethics Committee of Shanghai Sixth People’s Hospital (No. 2019-134). All aspects of the study were performed in accordance with the Declaration of Helsinki. OA cartilage was harvested from the damaged area of arthritic cartilage layers, while undamaged cartilage harvested from non-arthritic cartilage acted as a control, as described in previous studies (15). All patients signed informed written consent forms. Tissue samples were frozen immediately in liquid nitrogen after isolation and kept frozen until use.

### RNA extraction and RT-PCR validation

Total RNA was extracted from cartilage tissue using a total RNA extraction kit (Shabio, Shanghai, China) in accordance with the manufacturer’s protocols. For circRNAs, total RNA was then digested with RNase R (Epicenter, Madison, WI, USA) and reverse transcribed into cDNA using NovoScript II reverse transcriptase (Novoprotein, Suzhou, China). RT-PCR was carried out using AceQ Universal SYBR qPCR Master Mix (Vazyme Biotech, Nanjing, China) in an ABI PRISM^®^ 7500 Sequence Detection System (Applied Biosystems Inc., Foster City, CA, USA). Replicate wells were set up for each sample. GAPDH was selected as the internal normalization control. Relative fold expression of circRNA was determined using the 2^−ΔΔct^ method. Primer sequences were synthesized by Sangon Biotech (Shanghai, China). The remaining cDNA and total RNA were dissolved in RNA Follow All solution (New Cell Biotech, Suzhou, China) and placed in a freezer at –80°C.

### Cell culture and *in vitro* OA model

Human C28/I2 chondrocytes were obtained from Immocell, China. The cells were obtained and cultured in DMEM/F12 medium containing 10% fetal bovine serum, 1% penicillin, and streptomycin (100 μg/ml). The cells were incubated at 37°C with 5% CO_2_. IL-1β, at a concentration of 10 ng/ml, was added to each group of cells to establish the *in vitro* OA model. Untreated cells were used as a control. siRNAs of hsa_circ_0027914, hsa_circ_0101125, and hsa_circ_0102564, made by GenePharma, were added to each well at a concentration of 50 nM and incubated for 24 hours.

### TUNEL staining

Th apoptosis level of chondrocytes was detected by using terminal deoxynucleotidyl transferase dUTP nick-end labeling (TUNEL) kits in accordance with the manufacturer’s protocol as previously described (16). Specially, the pretreated cell smears were fixed with 4% paraformaldehyde for 30 minutes and incubated with 20 μg/ml proteinase K for 10 minutes. Subsequently, cells were incubated with equilibration buffer solution for 30 minutes and freshly prepared TUNEL staining solution for 1 hour, avoiding exposure to light. Nuclei were stained with 4′,6-diamidino-2-phenylindole (DAPI) for 5 minutes. Randomly selected fields were captured under a fluorescence microscope (Leica, Germany).

### CCK-8 assay

The CCK-8 test was applied to detect the proliferation effect on chondrocytes treated with siRNA from the three hub DECs. Chondrocytes were seeded at a density of 5 × 10^3^ cells per well in 96-well plates for 3 consecutive days of testing. CCK-8 solution (10 μl per well) was added to each well and the plates incubated for 3 hours at 37°C following the manufacturer’s protocols. Absorbance was examined at 460 nm using a microplate reader (Thermo Fisher Scientific). All three trials were repeated three times to meet statistical requirements.

### Enzyme-linked immunosorbent assay

Cells were seeded at a density 1 × 10^6^ cells per well in six-well plates to detect the secretion levels of IL-6 and TNF-α. IL-1β and siRNAs from the three hub DECs were intervened when chondrocytes reached a confluence of 80%. Cell supernatant was harvested from each well and an ELISA kit was used to measure the concentrations of IL-6 and TNF-α following the manufacturer’s protocol as in a previous study (11). The absorbance was examined at 450 nm using a microplate reader (Thermo Fisher Scientific). Triplicate experiments were used to obtain the mean value.

### Statistical analysis

Statistical analysis was performed using SPSS 16.0 software. Data are shown as means ± SDs. Statistical differences between two groups were compared using the independent-samples *t*-test. The threshold of statistical difference was set at a *p*-value < 0.05.

## Results

Bioinformatics, being a convenient, high-throughput, and predictably accurate tool, is increasingly used by researchers, especially in the study of degenerative diseases such as OA. Gene networks have been widely used to screen pathogenic genes, to identify clinical biomarkers, to elucidate epigenetic regulatory mechanisms, and to identify potential new drug targets for future experimental verification (17). In this study, novel pathogenic circRNAs and their regulation of microRNAs and genes were identified as playing a role in OA pathogenesis. Drugs targeting these genes were screened to identify those with the greatest affinity for these genes.

### Identification of DEGs, DEMs, and DECs

Based on the above-mentioned threshold, differentially expressed genes and microRNAs were selected from four GEO datasets, as shown in **Table 1**. Before screening DEGs in OA, the two independent expression datasets of mRNA processed on different platforms were normalized. The features of four GEO datasets are exhibited as a three-line watch in **Table 1**. In total, 2,886 DEGs, 2,261 DEGs, 76 DEMs, and 43 DECs were identified in GSE114007, GSE168505, GSE79258, and GSE175959. Volcano plots of GSE17959 (**Figure 1A**) and GSE79258 (**Figure 1B**) show the overall upregulated and downregulated DECs, and DEMs in arthritic cartilage and non-arthritic cartilage. Heatmaps (**Figures 1C, D**) and volcano plots (**Figures 1E, F**) of GSE114007 and GSE168505 show the distribution of upregulated and downregulated DEGs. The differentially expressed *p*-values of hub genes in GSE114007 and GSE168505 databases are listed in **Table 2**.** **A Venn diagram (**Figure 1G**) of the mRNA datasets overlapping GSE114007 and GSE168505 shows a total of 441 candidate DEGs. A total of 4,265 DEGs with opposite expression trends in these two datasets were excluded. After removing inconsistent expression in both datasets, the remaining 427 DEGs, comprising 367 upregulated DEGS and 60 downregulated DEGs, were considered candidate DEGs.

**Table 1:** Characterization of the four datasets from the GEO database.

Accession no.	Platform	Sample	Non-arthritic cartilage	Osteoarthritic cartilage	circRNA/microRNA/mRNA
GSE175959	GPL21825	Tissues	3	3	circRNA
GSE79258	GPL21599	Tissues	2	2	microRNA
GSE114007	GPL11154/GPL18573	Tissues	18	20	mRNA
GSE168505	GPL16791	Tissues	3	4	mRNA

**Table 2:** Differentially expressed *p*-values of hub genes in the GSE114007 and GSE168505 databases.

Gene	GSE114007 *p*-value	GSE16850 *p*-value	Gene	GSE114007 *p*-value	GSE16850 *p*-value
BIRC5	1.25479E-05	0.01481	DTL	0.004593409	0.020483
ZWINT	1.61251E-05	0.002706	EXO1	0.009738283	0.023251
CCNA2	1.22067E-05	0.014811	NEK2	0.01498041	0.028499
MCM10	0.023361068	0.02301	CEP55	0.000194963	0.030783
ASPM	2.01952E-09	0.027809	CDC25C	0.014188917	0.042123
ANLN	1.32865E-06	0.028623	CDC45	0.006994477	0.017533
CKAP2L	0.000236529	0.037765	ECT2	2.1904E-05	0.044262
PARPBP	0.008837195	0.013339	RRM2	2.84222E-05	0.03409
SKA1	0.001319427	0.048829	KIF23	1.77077E-06	0.022666
DEPDC1	1.29692E-05	0.018933	PLK1	0.000596262	0.043381
AURKB	0.001141327	0.045063	SHCBP1	0.001245922	0.019325
AURKA	1.39417E-05	0.049788	HMMR	0.000200977	0.018348
TTK	3.14754E-05	0.00073	CENPK	0.000197986	0.018214
FAM64A	0.008869907	0.002268	KIF15	0.002073268	0.044941
CDKN3	0.000181842	0.015816	NUSAP1	8.08765E-05	0.012801
BUB1	3.47034E-05	0.025316	NCAPG	8.82967E-05	0.020624
EZH2	4.52304E-06	0.003262	TOP2A	3.61644E-07	0.013508
MELK	4.05136E-05	0.027474	CLSPN	0.000683585	0.037467
KIF14	0.011879799	0.038972	CDK1	4.16892E-05	0.010887
ARHGAP11A	0.004992581	0.004564	KIF20A	4.21525E-05	0.003068
PBK	5.09596E-06	0.021727	BUB1B	0.00010006	0.027953
PTTG1	0.001645643	0.013477	CENPN	1.92797E-06	0.006739
ESCO2	0.047210258	0.020369	NDC80	0.010964047	0.009849
KPNA2	0.000819045	0.035039	UHRF1	0.002325478	0.012213
CDCA8	0.018665118	0.028464	PLK4	0.001191517	0.018608
KIF18A	0.021003146	0.031329	KIF4A	9.43291E-05	0.004651
UBE2C	0.004858359	0.007103	UBE2T	0.010003861	0.004464
NUF2	2.21472E-05	0.035093	CDC20	0.00203521	0.037308

### Construction of a PPI network and identification of hub genes

**Figure 2:**
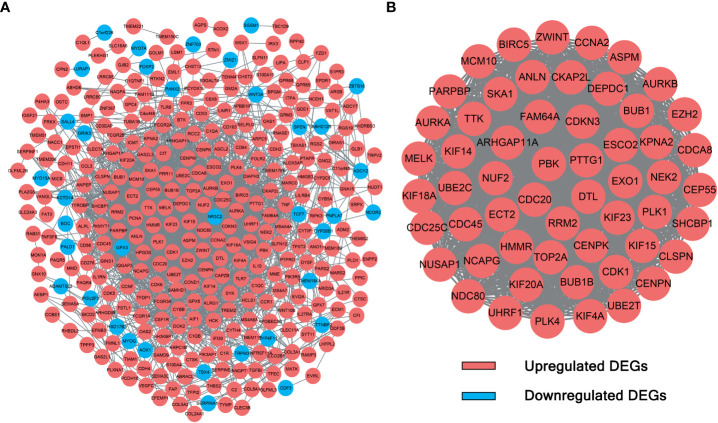
PPI network and module analysis. **(A)** The 339 DEGs in the PPI network. **(B)** Hub DEGs identified by module analysis using MCODE.

The interactions between candidate DEGs were determined by constructing a PPI network using the STRING tool. The results were visualized using Cytoscape, as shown in **Figure 2**. The PPI network was composed of 339 nodes (genes) and 2,854 edges (interactions). Furthermore, hub DEGs were identified by MCODE. As shown in **Figure 2**, the highest-rated module comprised 56 nodes (genes) and 1,452 edges (interactions). Interestingly, the hub DEGs were all upregulated genes.

### Enrichment analysis of DEGs and hub genes

**Figure 3:**
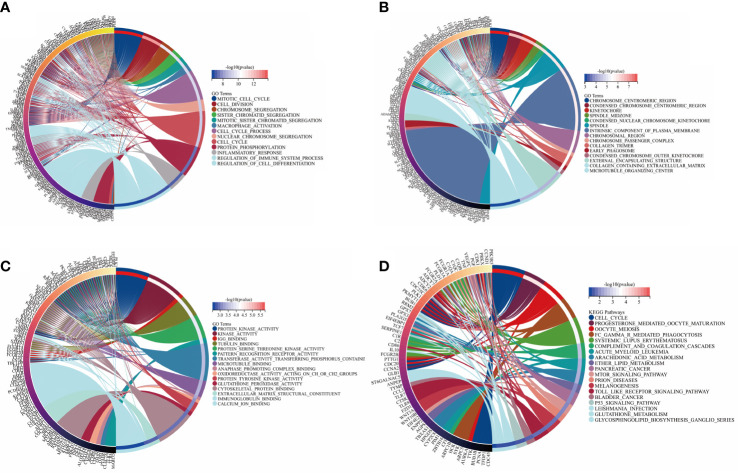
GO annotation and KEGG analysis of DEGs. **(A)** BP aspect; **(B)** CC aspect; **(C)** MF aspect; and **(D)** KEGG analysis.

**Figure 4:**
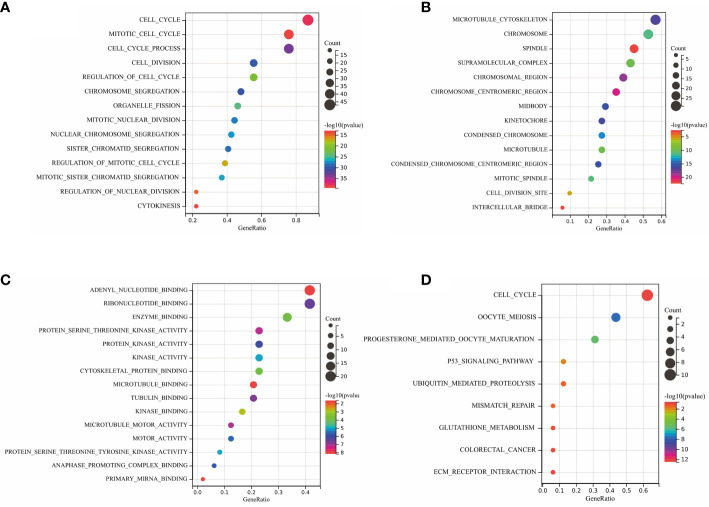
GO annotation and KEGG analysis of hub DEGs. **(A)** BP aspects; **(B)** CC aspects; **(C)** MF aspects; and **(D)** KEGG analysis.

To investigate the biological function and pathways of DEGs, KEGG and GO analyses were carried out on the 427 DEGs. The results of KEGG and GO analyses of hub genes and the related *p*-values are provided in **Supplementary Material 2**. **Figures 3A–C** demonstrates the mainly enriched functional annotations from three aspects: the biological process (BP), the cellular components (CCs), and the molecular functions (MFs). From the perspective of the BP, DEGs were mainly enriched during the mitotic cell cycle, cell division, and macrophage activation. From the perspective of CCs, DEGs were mainly involved in the chromosome centromeric region, the kinetochore, and the collagen-containing ECM. From the perspective of MFs, DEGs were mainly focused on protein kinase activity, tubulin binding, oxidoreductase activity, and ECM structural constituents. In addition, the KEGG analysis in **Figure 3D** shows that DEGs mainly participated in Fc𝛾 R-mediated phagocytosis, arachidonic acid metabolism, the mTOR pathway, and p53 pathways. In the case of the hub DEGs shown in **Figures 4A–D**, the BP items enriched were the mitotic cell cycle and cell cycle process; the CC items enriched were microtubule skeleton modulation and supramolecular complexes; and MF items enriched were cytoskeletal protein binding and protein kinase activity. KEGG results were involved in the p53 signaling pathway and ECM–receptor interaction.

### Drug–gene networks

**Figure 5:**
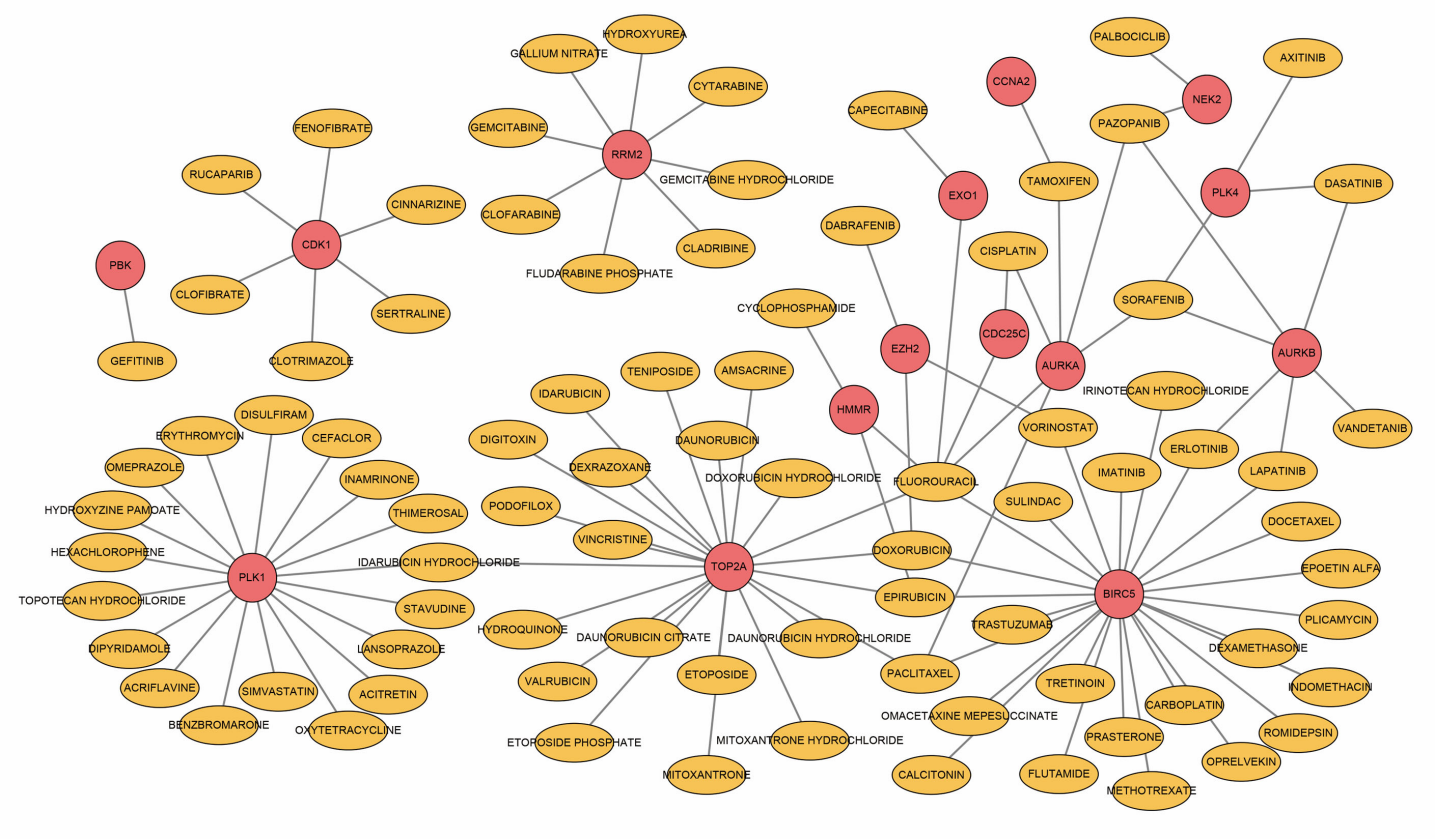
Drug–hub DEGs interplay network. Yellow circles, potential target drugs; red circles, upregulated DEGs.

To obtain target drugs for hub DEGs, drug–gene interactions were collected from the DGIdb database; 86 potential drugs for OA were identified. The results were visualized using Cytoscape, as shown in **Figure 5**. However, the underlying mechanism of drug–gene interaction could not be determined.

### Molecular docking

**Figure 6:**
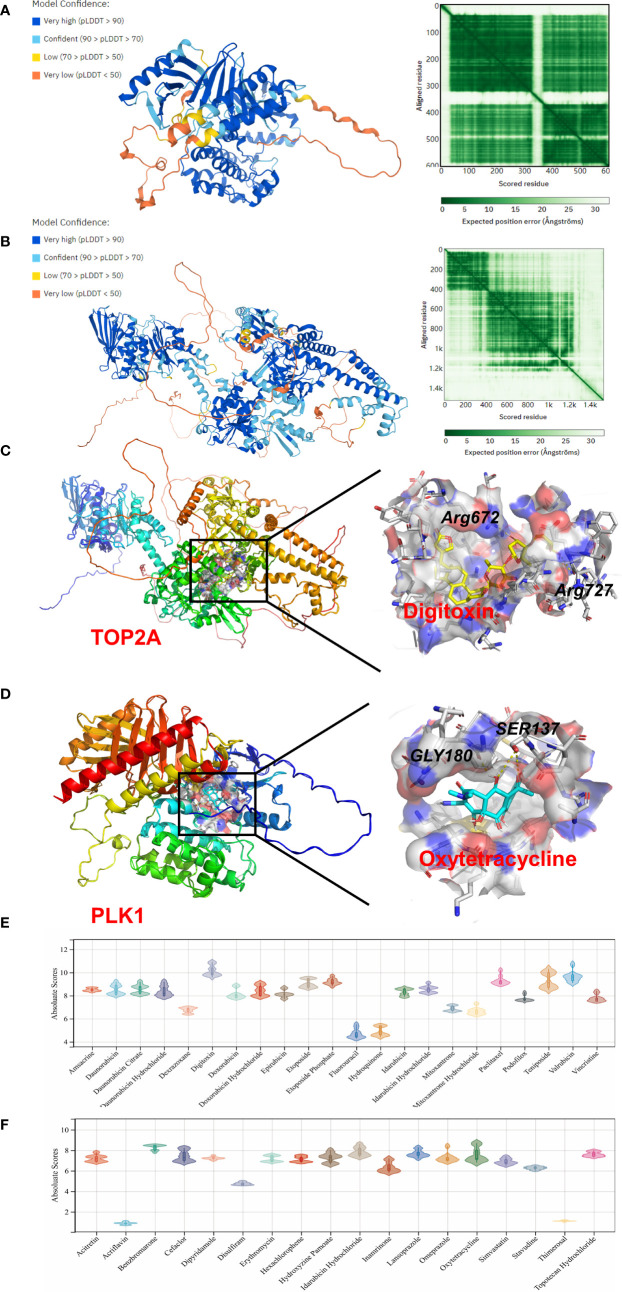
Homology modeling and molecular docking results. The crystal structure and evaluation of **(A)** TOP2A and **(B)** PLK1. The best docking positions **(C)** between digitoxin and TOP2A or **(D)** between oxytetracycline and PLK1 are indicated. The absolute value of affinity between predicated small molecules and **(E)** TOP2A or **(F)** PLK1 was exhibited.

To further investigate the molecular mechanism of drug–gene interaction, molecular docking was performed to identify the drugs with the most potential. The crystal structure of human TOP2A and PLK1 was predicted by AlphaFold v2.0. The LDDT score showed a benign stereochemical property of the predicted structures. The affinity score of AutoDock Vina was applied to evaluate the merits of docking. The scores of the potential drugs for hub DEGs are shown in **Figure 6**. The results indicate that digitoxin has the strongest binding affinity for TOP2A (0.0–0.0 kcal/mol, |interval score|) and oxytetracycline for PLK1 (0.0–0.0 kcal/mol, |interval score|). The perfect conformation showed that digitoxin interacts with residues of TOP2A (Arg672 and Arg727) through hydrogen bonds. Similar results were observed in the case of oxytetracycline, which interacts with residues of PLK1 (Ser137 and Gly180). Thus, through drug screening, digitoxin and oxytetracycline were found to have potential therapeutic effect in OA.

### Construction of the circRNA–miRNA–mRNA regulatory network

**Figure 7:**
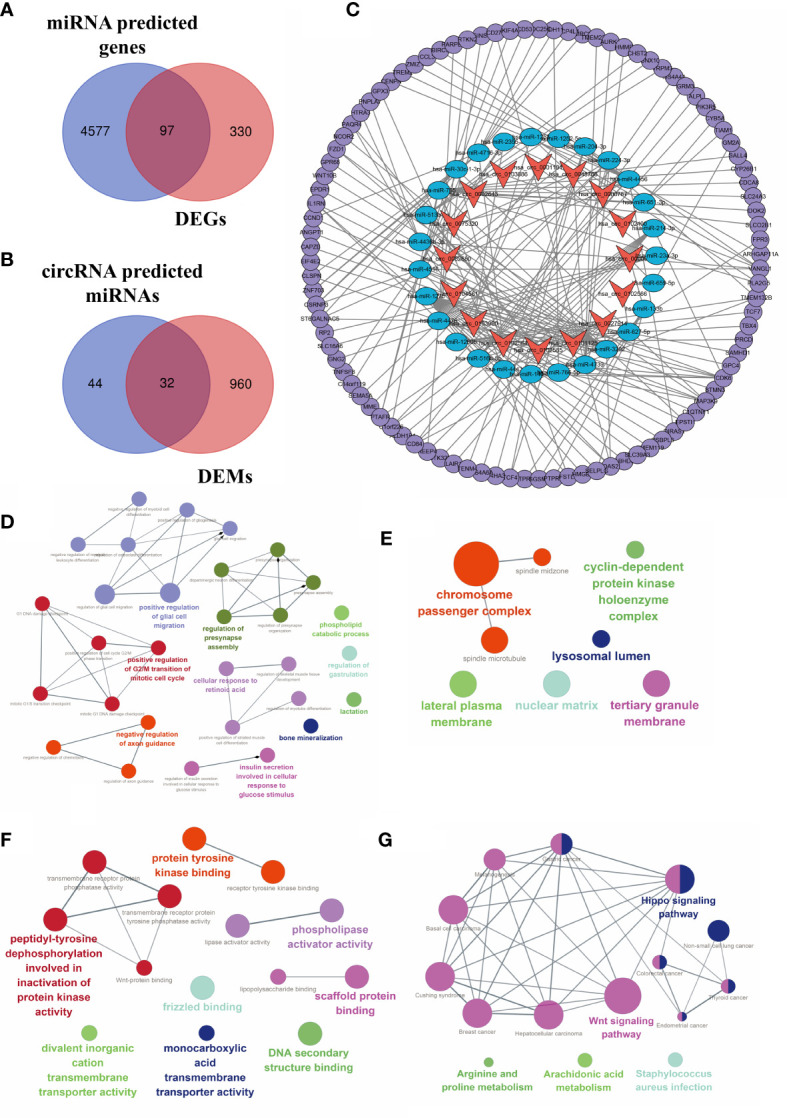
Construction of the circRNA–miRNA–mRNA regulatory network. **(A)** Venn diagram of the intersection of DEMs and predicted target genes. **(B)** The intersection of DECs and predicted target miRNAs. **(C)** The circRNA–miRNA–mRNA regulatory network. Orange arrows, DECs; blue ellipses, DETMs; purple circles, DETGs. Function annotation and KEGG analysis of hub DECs. **(D)** BP aspects; **(E)** CC aspects; **(F)** MF aspects; and **(G)** KEGG pathways.

CircBank was applied to predict target miRNA of DECs. A total of 76 miRNA genes predicted by circRNAs intersected with 992 DEMs. The 32 overlapping miRNAs were identified as DETMs. miRWalk 3.0 was applied to predict target genes of miRNAs. In our study, a total of 4,674 genes predicted by DETMs intersected with 427 DEGs. The 97 overlapping genes were identified as DETGs for further analysis. We then integrated the circRNA–miRNA network and the miRNA–mRNA network and used Cytoscape to construct an entire regulatory circRNA–miRNA–mRNA network, as shown in **Figure 7**.

### Identification of hub DECs

Based on the established circRNA–miRNA–mRNA regulatory network, three hub DECs were selected based on the top three regulated target miRNAs. These three hub DECs were hsa_circ_0027914, hsa_circ_0101125, and hsa_circ_0102564.

### Functional enrichment analysis of hub DECs

Functional annotation of GO analysis and pathway enrichment of KEGG analysis were carried out to further explore the function of hub DECs, as illustrated in **Figure 7**. Three hub DECs were enriched in regulation of G_2_/M transition of the mitotic cell cycle, axon guidance, bone mineralization, and insulin metabolism, in BP terms; cyclin-dependent protein kinase holoenzyme complex, chromosome passenger complex, and lysosomal lumen, in CC terms; and scaffold protein binding, tyrosine protein kinase binding, and phospholipase activator activity, in MF terms. In addition, hub DECs were found to be involved in the Wnt and Hippo signaling pathway, arachidonic acid metabolism, and *Staphylococcus* infection, based on KEGG analysis.

### Validation of DECs with RT-PCR

**Figure 8:**
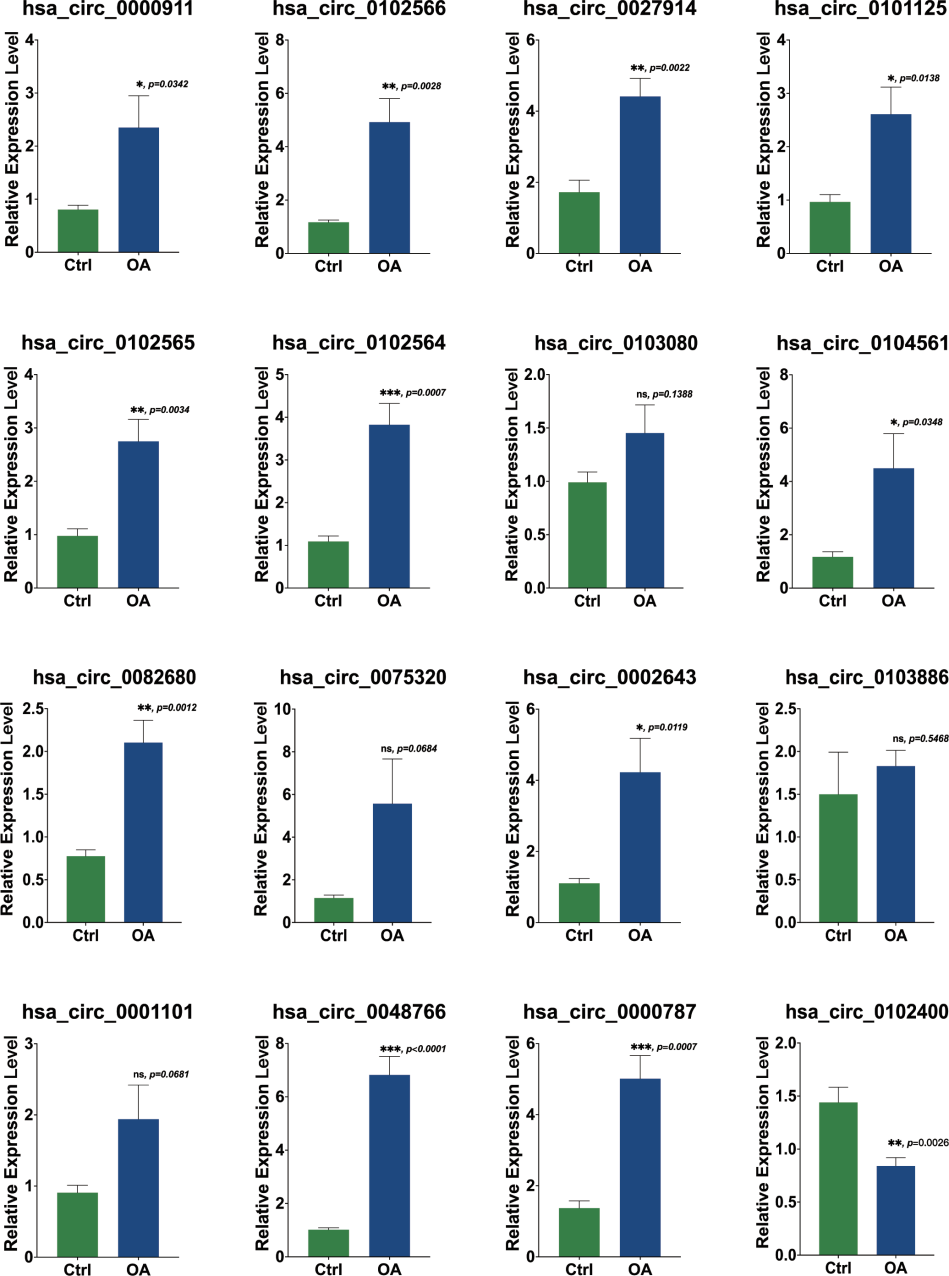
Validation of the expression of 16 DECs using RT-PCR. The relative expression levels of hsa_circ_00009, hsa_circ_0102566, hsa_circ_0027914, hsa_circ_0101125, hsa_circ_0102565, hsa_circ_0102564, hsa_circ_0104561, hsa_circ_0082680, hsa_circ_0002643, hsa_circ_0000787, and hsa_circ_0048766 were upregulated in OA cartilage, while the expression levels of hsa_circ_0103080, hsa_circ_0075320, hsa_circ_0001101, and hsa_circ_0103886 were not statistically significantly different. The relative expression level of hsa_circ_0102400 was obviously downregulated. Statistical difference: ***p < 0.001; **p < 0.01; *p < 0.05; ns*, p > *0.05.

To validate DECs expression in OA, RT-PCR was carried out on OA cartilage and non-arthritic cartilage (*n* = 5). Most of the results were consistent with our analysis. Eleven DECs were upregulated to a statistically significant degree. In the case of four other DECs, differences were not statistically significant. Furthermore, hsa_circ_0102400 was downregulated in our experiments, as shown in **Figure 8**. All three hub DECs were, as predicted, markedly upregulated in OA cartilage compared with non-arthritic cartilage.

### *In vitro* verification of functions of hub DECs

**Figure 9:**
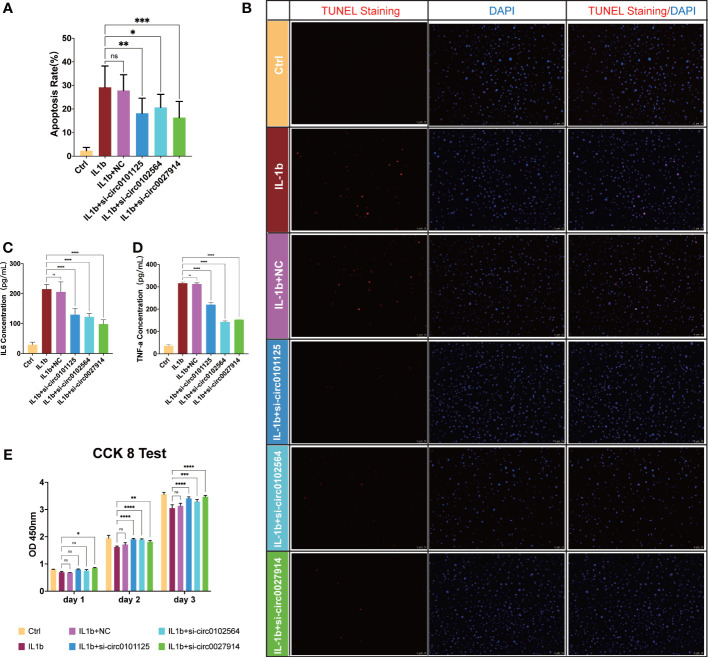
Verification of the function of the three hub DECs in an *in vitro* IL-1β-mediated OA chondrocyte model. Representative figures of TUNEL staining **(B)** and quantitative analysis **(A)** demonstrated the alleviation of apoptosis chondrocytes in siRNAs from three hub DECs. ELISA of IL-6 **(C)** and TNF-α **(D)** demonstrated the inhibition of inflammatory cytokine secretion with silencing intervention of these three DECs. **(E)** The CCK-8 test demonstrated that proliferation of IL-1β-treated chondrocytes was restored by silencing these three hub DECs on 1, 2, or 3 days. ****p < 0.0001; ***p < 0.001; **p < 0.01; *p < 0.05*;* ns, *p > *0.05.

*In vitro* IL-1β-mediated OA chondrocyte models were established to verify the intervention of these three hub DECs through siRNAs. SiRNA interference efficiency has been verified, as shown in **Figure S1**. TUNEL staining (**Figures 9A, B**) showed a statistically significant difference in the extent to which apoptosis of chondrocytes was reduced by siRNAs in different hub DECs. ELISA of IL-6 (**Figure 9C**) and TNF-α (**Figure 9D**) demonstrated the inhibition of inflammatory cytokine secretion with silencing intervention of these three DECs. The CCK-8 test demonstrated that proliferation of IL-1β-treated chondrocytes was restored by silencing these three hub DECs for 1, 2, and 3 days (**Figure 9E**). Our preliminary results indicated that silencing of hsa_circ_0027914 in the three hub DECs had the desired effect of reducing IL-1β-mediated apoptosis of OA chondrocytes.

## Discussion

Osteoarthritis is the most common degenerative orthopedic disease, and the prevalence of osteoarthritis increases with age in all joints of the body. In contrast to other diseases, OA is not associated with a high mortality rate, but it is a chronic, progressive disease that adversely affects patients’ quality of life. However, a lack of understanding of the biological mechanisms underlying the disease etiology and of existing therapeutic strategies for preventing or treating cartilage destruction or for reconstructing the subchondral bone are limited. Symptom-only medications are often rejected by patients for fear of long-term dependence. Therefore, OA drug treatment cannot be limited to symptom relief, and should instead focus on new drug targets based on the latest results of genetic research, which indicates that screening of hub ncRNAs and mRNAs associated with OA is urgently needed. The identification of previously undiscovered ncRNAs would enable the occurrence and development of OA to be recognized at an earlier clinical stage than at present. The prevention of OA or its early diagnosis would improve the prognosis of patients and reduce the need for surgery. Yet, to our knowledge, there have been no reports establishing circRNA–miRNA–mRNA regulatory networks in OA. We have revealed, for the first time, internal interactions among circRNAs, miRNAs, and mRNAs and we revisit OA therapy from a novel angle.

We screened out 427 DEGs by overlapping two mRNA datasets. The results of enrichment analysis of DEGs were closely related to mitotic cell cycle, cell division, and macrophage activation, all of which are closely associated with the pathogenesis of OA, including cartilage and subchondral destruction and synovial inflammation. KEGG results also indicated that DEGs are mainly associated with cell proliferation, apoptosis, and ECM structural constituent. In addition, many ECM structural protein genes, such as *COL24A1* and *COL3A1*, were found in DEGs, which is consistent with widespread destruction of cartilage (18).

To screen out the hub DEGs, 56 hub DEGs were all upregulated as shown in the PPI network. Although most of these hub DEGs were not directly associated with OA, these data are also consistent with the pathological changes in OA. From the perspective of bone and cartilage changes, CDC20 and TOP2A, which are related to cancer cell proliferation and progression, were recently suggested to play a role in rat bone marrow stem cell osteogenesis and chondrogenesis (19). In chondrocytes, upregulation of CDC20 and TOP2A might be an indication of insufficient compensatory cartilage and bone regeneration capacity in response to the destruction of cartilage and subchondral bone. Furthermore, MCM10, a highly conserved pre-replication complex, was recently reported to be a modulator of DNA replication timing (20), suggesting that it plays a potential regulatory role in chondrocyte survival in OA at a deeper genetic level. In addition, another important aspect of OA is the synovial change (21). IL-1RN is one of the cytokines implicated in OA at the inflammation level (22). It has been reported that the IL-1–IL-17 signaling axis induces cartilage destruction and OA development by enhancing the expression of catabolic factors (23). More importantly, IL-1RN polymorphism has an interesting clinical use. The TTG haplotype of IL-1RN is associated with radiographically more severe OA and an increased risk for incident OA (24). In addition, CD53, a molecule vital to lymphocyte trafficking and immunity (25), has been found to play an essential role in OA progression (26). Hence, most of the results of the microarray analysis were in accordance with previous studies and provided supportive evidence for further analysis.

Although many oral pain relief medications, such as non-steroidal anti-inflammatory drugs (NSAIDs), are widely used in the early treatment of OA, they can only treat symptoms owing to the lack of a well-defined target and are always associated with side-effects (for instance, gastrointestinal bleeding in the case of NSAIDs). If oral drugs are ineffective, the injection of prednisone or anesthetic drugs or joint replacement surgery is necessary to relieve patient suffering. Therefore, target drugs for OA urgently need to be developed to avoid progression of degeneration of the joint. Based on our predicted hub DEGs, 86 drugs approved by the Food and Drug Administration of the USA (FDA) were discovered through DGIdb database. Given the top three ranking of target drugs for hub DEGs, TOP2A, PLK1, and BIRC5, computational molecular docking was applied to identify the best candidates. As shown in previous studies, irinotecan has the strongest binding to BIRC5 through hydrogen bond formation (12). In this study, digitoxin and oxytetracycline were found to have the greatest binding affinity for TOP2A and PLK1, respectively. Digitoxin is a cardiac glycoside used in the treatment of congestive heart failure. Cardiac glycosides, such as frugoside, possess interesting anti-inflammatory potential, and can delay OA progression through inhibition of synovial inflammation (27). Oxytetracycline, in contrast, is a tetracycline antibiotic effective against Gram-positive bacteria. Oxytetracycline has already been identified as a chondrogenic compound and a potential OA treatment drug, stimulating cartilage regeneration, as described in a previous study (28). These two drugs are commonly used in clinical practice, and results of the molecular docking experiments might prove to be of medical relevance for the treatment of OA. We expect that our results could provide more insights into OA and potential therapeutic targets.

Over the last few decades, the function of circRNAs has become one of the most discussed issues, and circRNAs have been investigated as novel biomarkers of multiple diseases. Accumulated evidence indicates that circRNAs function as a sponge for the miRNAs involved in post-transcriptional regulation in OA (29). A recent study investigated the circRNA profiles in the osteoarthritic synovium and found that the hsa_circ_0072697–hsa_miR-6736–5p-LEP/ULK1 axis plays a role in cell senescence regulation (30). In our study, we constructed a network of 16 DECs, regulated miRNAs, and target mRNAs. hsa_circ_0027914, hsa_circ_0101125, and hsa_circ_0102564 were identified as hub DECs based on the number of miRNAs regulated. Functional annotation analysis of hub DECs indicated that DECs are involved in regulation of mitotic cell cycle, axon guidance, bone mineralization, protein kinase binding, etc. The axon guidance suggested the nerve-regulated potential of bone metastasis, which is consistent with previous studies indicating that prostaglandin (PG) E_2_-mediated sensory nerve regulation has a role in bone homeostasis (31). Pathway enrichment analysis revealed the involvement of the Wnt and Hippo signaling pathways and arachidonic acid metabolism in OA pathogenesis. Interestingly, a previous study found that the arachidonic acid metabolite prostaglandin D2 (PGD^2^) regulates polo-like kinase 1 (PLK1) and mediates chondrocyte apoptosis (32), which is in accordance with our results. Other processes, including bone mineralization and insulin metabolism, have also been closely linked with pathological symptoms in OA, such as the destruction of cartilage and subchondral bone.

Among the 16 DECs, a role has been elucidated only for hsa_circ_0000787, which has been detected in the peripheral blood of patients with type 1 diabetes mellitus (33) and systemic lupus erythematosus (34). However, there have been no studies on its functions or its role in OA. OA pathogenesis of fibrosis circRNA-related studies have been performed on circ_0000423. High levels of expression of circ_0000423 in OA regulate miRNA-27b-3p, and increased levels of the chondrocyte hypertrophy marker MMP-13 have been detected in OA cartilage. Application of AAV-shRNA-circ_0000423 slows the progression of OA by decreasing joint surface fibrosis and osteophyte formation (35). In addition, circHIPK3 (circBase 000284), derived from mesenchymal stem cells, regulates miR-124-3p and MYH9 to prevent chondrocyte apoptosis and hypertrophy in the IL-1β-induced osteoarthritis model (36).

Owing to a lack of experimental studies, we can only speculate on the functions of our novel DECs, based on the functions of the miRNAs or mRNAs they regulate.

hsa_circ_0027914, one of the hub DECs, regulated the greatest number of miRNAs, including hsa-miR-140-3p, hsa-miR-766-5p, hsa-miR-1260b, hsa-miR-516b-5p, hsa-miR-4443, hsa-miR-4739, and hsa-miR-3202. Of these, hsa-miR-140-3p and hsa-miR-766-5p (37) have been reported to participate in the mechanism of OA. MiR-140, the expression of which is especially low in OA cartilage (38), has been recently considered as a promising therapeutic strategy in OA (39). The underlying mechanism might be due to target genes of miR-140, including *ADAMTS5* (40), *MMP13* (41), *CXCR4* (42), *IGFBP5* (43), etc. These target genes are essential regulating genes in cartilage homeostasis, including cartilage degradation and inflammation and cellular fibrosis and hypertrophy in OA (9). Nevertheless, target DEGs in our study mainly focus on cell apoptosis and proliferation. Hence, hsa_circ_0027914 might down-regulate miR-140-3p through a competitive endogenous mechanism and lead to upregulation of DEGs in OA. A recent study has also reported that another circRNA (i.e., hsa_circ_0104595) can sponge miR-140-3p, then target EZH2 (enhancer of zeste homolog 2) to induce apoptosis of chondrocytes in OA (44). Furthermore, when the effect of si-circ-0027914 in reducing apoptosis is taken into consideration, there are reasons to believe that hsa_circ_0027914 plays a vital role in regulating multiple miRNAs and the progression of OA.

Another hub DEC, hsa_circ_0101125, regulated fewer miRNAs, including hsa-miR-3202, hsa-miR-4516, hsa-miR-1275, and hsa-miR-4476. However, the functions of hsa-miR-3202, hsa-miR-4516, and hsa-miR-4476 in OA have not been investigated. According to previous studies in other fields, miR-3202 can promote endothelial cell apoptosis through FAIM (45); miR-4516 inhibits liver cancer cell proliferation and progression through SOX5 (46); miR-4476 could promote glioma progression through APC/β-catenin in the Wnt pathway (47). From the perspective of regulated DEGs, *CCND1* was one of DEGs regulated by miR-3202, according to our analysis. Considering the analogous results of CCND1 regulated by miR-142-5p in chondrocytes, our results suggest that miR-3202/CCND1 may also participate in chondrocyte autophagy and proliferation in OA (48). Hence, in combination with the regulatory role of miR-1275/MMP13 in chondrogenesis (49), it is reasonable to infer that hsa_circ_0101125 might regulate chondrocyte proliferation, migration, and chondrogenesis in OA.

The other hub DEC, hsa_circ_0102564, regulated four miRNAs: hsa-miR-766-5p, hsa-miR-765, hsa-miR-30c-1-3p, and hsa-miR-4716-3p. Owing to the intricated regulated network, miR-766 was also regulated by hsa_circ_0027914, as discussed above. MiR-30c has also recently been considered as a suppressor of bone gamma-carboxyglutamate protein (*BGLAP*) in osteogenesis (50). Target DEGs of miR-30c in our analysis were mostly related to the anti-apoptosis of abnormal tumor cells, including MAP3K9 in lung cancer (46), ST6GALNAC5 in prostate cancer (51), CSRNP3 in clear renal cell carcinoma (52), ZNF703 in breast tumors (53), and CLSPN in brain tumors (54). Thus, upregulation of hsa_circ_0102564 might help reveal the downstream mechanism involved in the abnormal maturation and proliferation of chondrocytes in OA.

In addition to speculating on the biological functions of hub DECs and related DETMs, we found that the role of several other miRNAs in OA pathogenesis is correlated with cell proliferation, migration, and ECM synthesis. For instance, miR-23a-3p, regulated by hsa_circ_0000911, has been found to be an essential regulator of SMAD3/TGF-β in cartilage senescence in OA (55, 56). miR-659-5p, which is regulated by hsa_circ_0000911, despite a lack of osteoarthritis-related studies so far, is an emerging target of the MAPK pathway in breast cancer (57). miR-133b, a cell proliferation regulator, has also been shown to be associated with carcinogenesis (58). The expression of both miR-140-3p and miR-627-5p has been shown to be significantly different in rheumatoid arthritis patients and control subjects, and it is thought that they are mainly involved in the cell proliferation process (59).

To investigate the competing endogenous effect between circRNA and miRNA, a circRNA–miRNA–mRNA regulatory network was established and 16 DECs and 32 DETMs were verified to be coregulators of 97 DETGs. Owing to the complex regulatory mechanism of circRNAs and miRNAs, the complicated network provided a novel insight into the competing endogenous RNA interplay between circRNAs and miRNAs. The three hub DECs could also be potential candidate biomarkers in OA treatment.

RT-PCR was performed to validate expression level of DECs in osteoarthritic cartilages. In comparison with non-arthritic cartilage, expression levels of 11 circRNAs were upregulated and the expression of circRNA was downregulated, as predicted in our analysis. No statistically significant differences in the expression levels of four circRNAs were found. Other *in vitro* studies involving TUNEL staining, the CCK-8 test, and ELISA of IL-6 and TNF-α have demonstrated a decrease in apoptosis following treatment with siRNA from these three circRNAs. CCK-8 showed that IL-1β-treated chondrocyte proliferation was increased by the intervention of hub DECs, especially the group of hsa_circ_0027914. ELISA results have also verified that inflammatory cytokines, including IL-6 and TNF-α, are reduced by the intervention of hub DECs.

Of course, there are inevitably some limitations in our research. Although we performed *in vitro* and *in vivo* experiments, the small sample size of the circRNA and miRNA datasets remains the main limitation of this study. Moreover, the function of downstream target genes and the predicted drugs were not investigated in this study. Thorough verification of the whole regulatory network should be the subject of future studies. Furthermore, because our biomarker was derived from cartilage, its diagnostic efficiency or related survival analysis could not be completed because of the lack of a worldwide comprehensive database. In addition, functional annotation and pathway enrichment analysis were conducted based on a tumor-related database, and thus our results are not necessarily applicable to OA-related mechanisms and processes. Thus, further studies will shed more light on the functional validation experiments and clinical translational research will be necessary to verify our results. Interaction between circRNA and miRNA is one of the potential biological underlying mechanisms. Whether other ncRNAs, such as lncRNA, also play an important role in OA remains worthy of discussion. We expect to discover more ncRNAs or ncRNAs diagnostic portfolio with high diagnostic efficiency to enhance the accuracy of early screening and improve patients’ prognosis.

## Conclusion

This is the first study to identify important biomarkers of circRNAs in OA. In addition, we shed light on the regulatory functions and potential molecular mechanisms of the circRNA–miRNA–mRNA network in OA pathogenesis.

## Data availability statement

The original contributions presented in the study are included in the article/**Supplementary Material**. Further enquiries can be directed to the corresponding authors.

## Ethics statement

The studies involving human participants were reviewed and approved by the Ethics Committee of Shanghai Sixth People’s hospital. The patients/participants provided their written informed consent to participate in this study.

## Author contributions

XL, SW, and GW designed this study. XL and HX administered the project and collected data. HX performed the experiments and analyzed these data. XP and SW conducted the bioinformatic analysis. XL wrote this manuscript. YC provided funding and resources. SW and GW modified and discussed this manuscript. All authors contributed to the article and approved the submitted version.
