# Supplementary material for: Identification and comprehensive analysis of circRNA–miRNA–mRNA regulatory networks in osteoarthritis
Source: Front Immunol. 2023 Jan 9;13:1050743. doi: 10.3389/fimmu.2022.1050743 (PMC9869167; doi:10.3389/fimmu.2022.1050743)
Supplement: Supplementary file 1 [file DataSheet_1.docx]

**Supplementary Information**

**Identification and comprehensive analysis of circRNA-miRNA-mRNA regulatory networks in osteoarthritis**

Xuanzhe Liu^1, †^, Huimin Xiao^2, †^, Xiaotong Peng^3, †^, Shuo Wang^1,^ ^*^, Gen Wen^1, *^

^1^Department of Orthopedic Surgery, Shanghai Jiao Tong University Affiliated Sixth People’s Hospital, Shanghai, 200233, China

^2^College of Fisheries and Life Science, Shanghai Ocean University, Shanghai, 201306, China

^3^Department of Gynaecology and Obstetrics, Xiangya Hospital, Central South University, Changsha, China,

^*^Corresponding authors:

Gen Wen, wengen2006@126.com

Shuo Wang, shuowang97@126.com

^†^The authors contributed equally to this work.

**SI Table 1 RNA sequences of siRNA of DECs and negative control**

|  | Sense(S)/Antisense (AS) | RNA sequence (5’-3’) |
| --- | --- | --- |
| Negative Control | S | UUCUCCGAACGUGUCACGUTT |
|  | AS | ACGUGACACGUUCGGAGAATT |
| si-circ-0027914 | S | GAAAAGGAGCCAGCGCGGUTT |
|  | AS | ACCGCGCUGGCUCCUUUUCTT |
| si-circ-0101125 | S | UCCAUUUCAACGAGCUUCUTT |
|  | AS | AGAAGCUCGUUGAAAUGGATT |
| si-circ-0102564 | S | UUUUGAGACAAGAGUGAGATT |
|  | AS | UCUCACUCUUGUCUCAAAATT |

**SI Table 2 RNA sequence of circRNAs were designed by Primer 3.0[1]**

|  | Forward(F)/Reverse(R) | RNA sequence (5’-3’) |
| --- | --- | --- |
| hsa_circ_0000911 | F | GGGGAAACTGAGTCATGGGA |
|  | R | AAGCTGAACCTGACCGAAGT |
| hsa_circ_0102566 | F | GACCTGCATGATGACGTTCG |
|  | R | ACATCCTTCTCACCCACCTG |
| hsa_circ_0027914 | F | TCCCCATGATTGTCTGTGCT |
|  | R | GTCGGGTTCCTTTCGCTTTT |
| hsa_circ_0101125 | F | TACCCCAAACATCGACGAGG |
|  | R | TGGAAGGCTGTTACCCGAAT |
| hsa_circ_0102565 | F | CTTTCTCCCGGATCAGTGGA |
|  | R | ACATCCTTCTCACCCACCTG |
| hsa_circ_0102564 | F | AGATGCTATCTCAAAGGAACAAAAT |
|  | R | TGCTACCAAAATTGTTTAAGAATTGTT |
| hsa_circ_0103080 | F | CCCTCAGACACCCACTAACA |
|  | R | CCATTACTTCAGCTCTGCCAC |
| hsa_circ_0104561 | F | TGCCAGACTGATTTCCGATTC |
|  | R | GCACACGACCACAATCCATT |
| hsa_circ_0082680 | F | CCACCTACTTGCAGTCCAGA |
|  | R | ATTGAAGGGTGTGAGGGGAG |
| hsa_circ_0075320 | F | CTCCAGTCCCTACAGATGCC |
|  | R | CACGTAGGACATGGCCATTG |
| hsa_circ_0002643 | F | CTTCGTGGATTTGGGGAGTG |
|  | R | TCAATGGAGGCTGTCAGGAG |
| hsa_circ_0103886 | F | TGGATCCACATATCTTTGCAGTA |
|  | R | CGTAAGTGAGGCAGCTCC |
| hsa_circ_0001101 | F | GGGACCCTATTCAGCGGTTA |
|  | R | TGTTCCACACTGCAATTCCG |
| hsa_circ_0048766 | F | AAGACTGAGCCCCTCCCC |
|  | R | ACACACCTCCCGAATCATGT |
| hsa_circ_0000787 | F | TCCACCAGAGCACCATAGAC |
|  | R | GCTGTTGACCCTCTTCTCCT |
| hsa_circ_0102400 | F | TAGAGGGCCAGATCGCAAAA |
|  | R | TCTGCCCAAAATGTCTCTCTG |

[1] Untergasser A, Cutcutache I, Koressaar T, Ye J, Faircloth BC, Remm M and Rozen SG.
Primer3--new capabilities and interfaces. Nucleic Acids Res. 2012 Aug 1;40(15):e115.

**SI Figure1 RNA inteferencing efficiency of DECs**


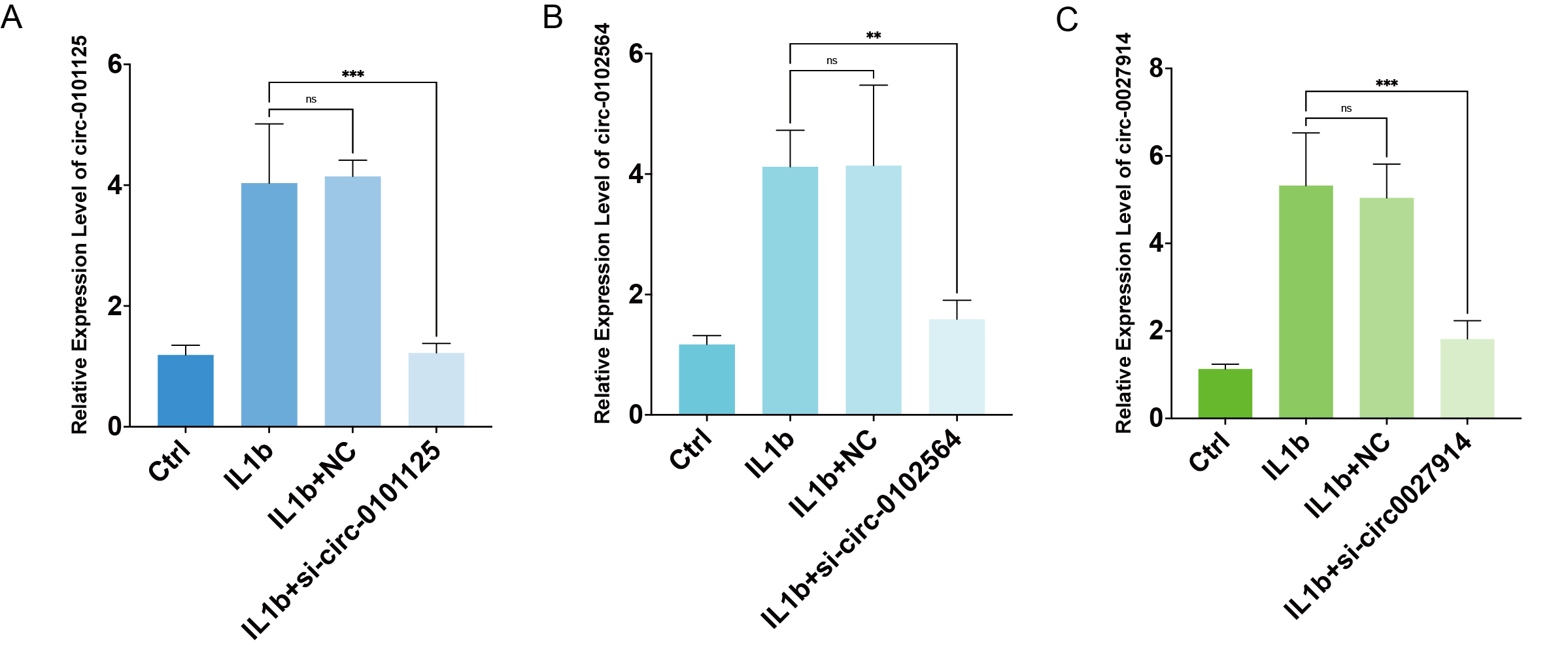


**Figure1 RNA inteferencing efficiency of DECs**. qPCR results have demonstrated the interference efficiency of siRNAs on circ-0101125(A), circ-0102564(B) and circ-0027914(C) in IL1b mediated in vitro OA chondrocytes.
